# Supplementary material for: Amino acids stimulate the endosome-to-Golgi trafficking through Ragulator and small GTPase Arl5
Source: Nat Commun. 2018 Nov 26;9:4987. doi: 10.1038/s41467-018-07444-y (PMC6255761; doi:10.1038/s41467-018-07444-y)
Supplement: Supplementary file 1 — Supplementary Information [file 41467_2018_7444_MOESM1_ESM.pdf]

Supplementary Information

Amino acids stimulate the endosome-to-Golgi trafficking through Ragulator and small GTPase Arl5

Shi et al.

Supplementary Figure 1

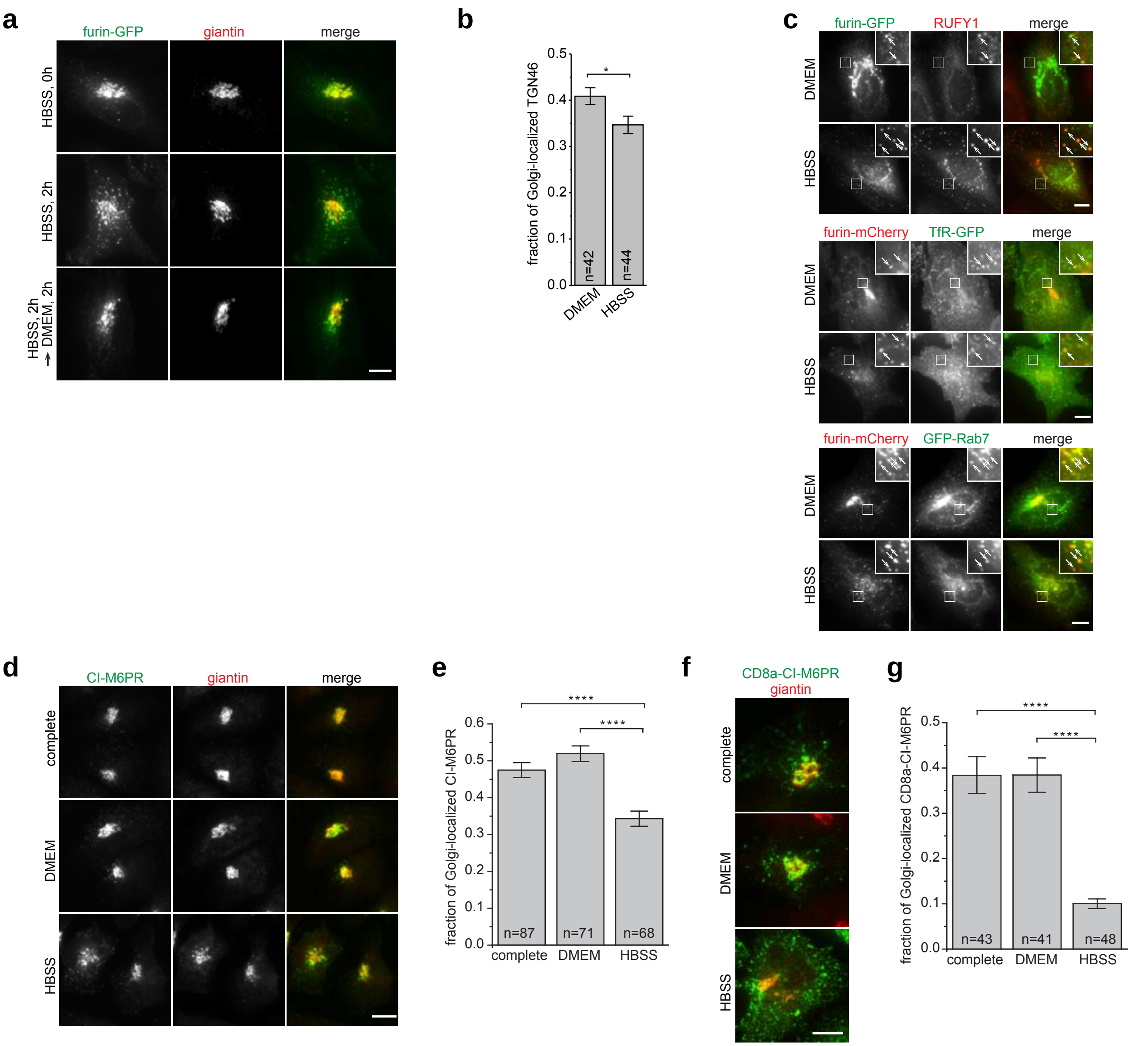

Supplementary Figure 1 Nutrient starvation induces endosomal translocation of TGN membrane proteins. All cells are HeLa cells. (**a**) The localization of furin-GFP during HBSS and DMEM treatment. Cells were processed as described in Fig. 1d. (**b**) The fraction of Golgi-localized TGN46 during HBSS and DMEM treatment. The experiment is similar to Fig. 1b. (**c**) The endosomal localization of furin-GFP or furin-mCherry increases at the expense of its Golgi pool during nutrient starvation. Cells expressing furin-GFP singly (top two panels), furin-mCherry together with TfR-GFP (middle two panels) and furin-mCherry together with GFP-Rab7 (lower two panels) were treated with indicated medium for 2 h. Cells in the top two panels were stained for endogenous RUFY1. Boxed regions are enlarged at the upper right corner to demonstrate the colocalization (indicated by arrows). (**d**,**e**) Nutrient starvation induces significant endosomal translocation of endogenous CI-M6PR. Cells were treated with indicated medium for 1 h and endogenous CI-M6PR and giantin were stained. The fraction of Golgi-localized CI-M6PR is quantified in e. (**f**,**g**) Nutrient starvation induces significant endosomal translocation of CD8a-CI-M6PR. The experiment was conducted similarly to **d** and **e** except that cells expressing CD8a-CI-M6PR were used and CD8a was stained. **b**, **e** and **g** are representative results from three independent experiments. Complete, complete medium; n, the number of cells analyzed; error bar, s.e.m.; scale bar, 10 μm; *P*-values were from *t*-test; *, $P \leq 0.05$; ****, $P \leq 0.00005$.

Supplementary Figure 2

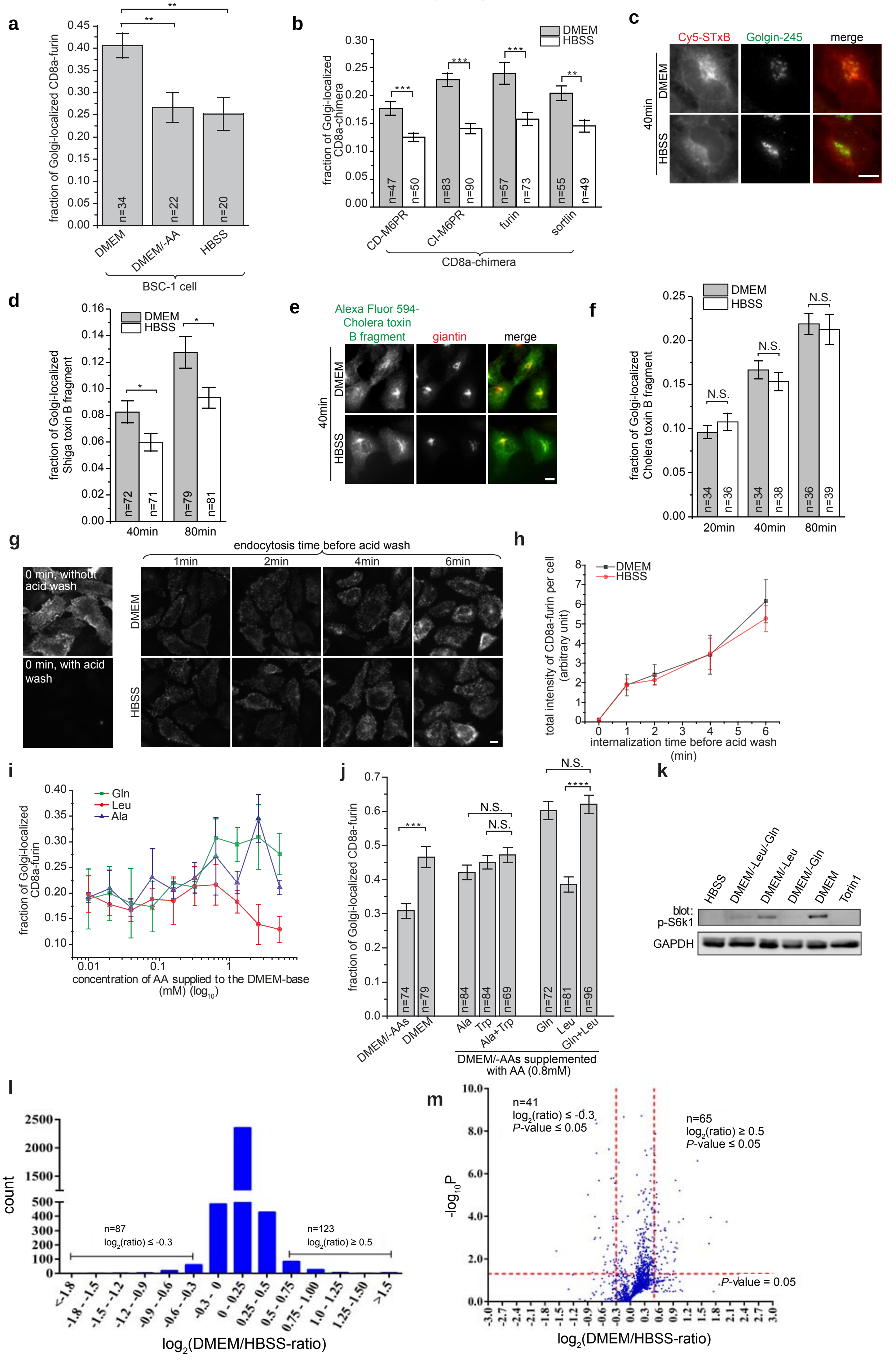

Supplementary Figure 2 AAs stimulate endocytic trafficking to the Golgi. (**a**) BSC-1 cells transiently expressing CD8a-furin were incubated in HBSS for 2 h followed by surface-labeling using anti-CD8a antibody. Cells were subsequently incubated in indicated medium for 20 min before immuno-labeling for CD8a and endogenous giantin. The fraction of the Golgi-localized CD8a-furin is quantified. (**b**) Similar to **a** except that HeLa cells transiently expressing indicated CD8a-fused chimeras were used. (**c-f)** AAs stimulate the endocytic trafficking of STxB but not Cholera toxin B fragment. HeLa cells were starved in HBSS for 2 h followed by incubation with indicated fluorophore-conjugated toxins. **c** and **e** are representative images while **d** and **f** are fractions of Golgi-localized toxins by quantitative imaging. (**g**,**h**) AAs do not significantly affect the endocytosis of CD8a-furin. HeLa cells expressing CD8a-furin were incubated in HBSS for 2 h followed by surface-labeling by anti-CD8a antibody. Next, labeled CD8a-furin was chased at 37 °C for indicated time before being subjected to acid wash on ice to remove the surface-bound antibody. Total cellular CD8a-furin was stained in **g** and the relative total CD8a intensity per cell was quantified in **h** as (total intensity of background-subtracted image)/(number of cells in the image). n=9 fields of view were used for each time point with each field having ≥ 10 cells. (**i**) HeLa cells stably expressing CD8a-furin were treated with DMEM/-AAs for 2 h followed by surface-labeling. The antibody-labeled CD8a-furin was subsequently chased for 20 min in DMEM/-AAs supplemented with indicated AA from 0.01 to 5.12 mM. After staining CD8a and giantin, cells were imaged and the Golgi-localized (I_{golgi}) and total CD8a-furin intensity (I_{total}) within each field of view were acquired. The fraction of Golgi-localized CD8a-furin is calculated as I_{golgi}/I_{total}. The plot incorporates n=4 (Ala and Gln) or 5 (Leu) randomly chosen fields of views with each field having ≥ 60 cells. (**j**) The effect of combining two AAs on the endocytic trafficking to the Golgi. Cells were treated as in **i** except with 0.8 mM indicated AAs. (**k**) Gln is essential for Leu to activate mTORC1 activity, as previously reported. HeLa cells were nutrient starved in HBSS for 2 h before treatment with indicated medium or Torin1 (in the complete medium) for 20 min. Endogenous phospho-S6K1 (p-S6K1) and GAPDH were blotted. **a**, **b**, **d**, **f**, **h**, **i** and **j** are representative results from three independent experiments. Error bar in **a**, **b**, **d**, **f** and **j**, s.e.m.; error bar in **h** and **i**, s.d.; n, the number of cells analyzed; scale bar, 10 µm; *P* -values were from t-test; N.S., not significant ($P > 0.05$); ∗, $P \leq 0.05$, ∗∗, $P \leq 0.005$; ∗∗∗, $P \leq 0.0005$; ∗∗∗∗, $P \leq 0.00005$. (**l**) Histogram of $\log_2$(DMEM/HBSS-ratio) of proteins identified from quantitative mass spectrometry. Heavy and light isotope labeled HeLa cells were treated with DMEM or HBSS and surface proteins were isolated and quantified by SILAC mass spectrometry. Forward and reverse labeling experiments were conducted with 3 replicates for each experiment. x axis, $\log_2$(DMEM/HBSS-ratio); y axis, the count of identified proteins. (**m**) Volcano plot to determine significantly changed proteins. x axis, $\log_2$(DMEM/HBSS-ratio); y axis, $-\log_{10}$(*P*-value). Statistical significance cut-off is $P \leq 0.05$. Upregulation cut-off for $\log_2$(DMEM/HBSS-ratio) is 0.5 while downregulation cut-off is -0.3.

Supplementary Figure 3

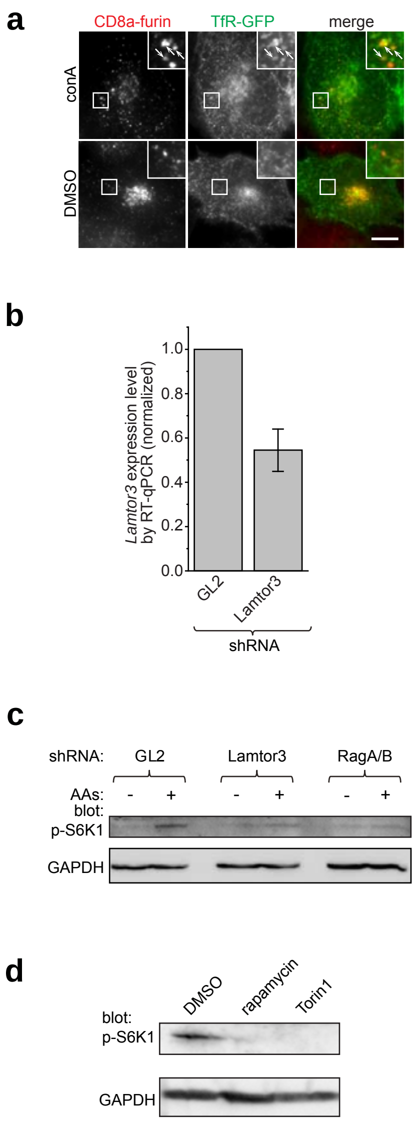

Supplementary Figure 3 This figure corresponds to Fig. 3. HeLa cells are used. (**a**) ConA induced the endosomal translocation of CD8a-furin. Cells co-expressing CD8a-furin and TfR-GFP were incubated in HBSS for 2 h followed by DMEM for 20 min. 1% DMSO or 2.5 μM conA was present throughout both HBSS and DMEM incubation. Boxed regions are enlarged at the upper right corner to demonstrate the colocalization (indicated by arrows). (**b**) Confirmation of the knockdown of Lamtor3. Cells were subjected to lentivirus-mediated knockdown using control shRNA (GL2) or shRNA targeting Lamtor3. The expression level of Lamtor3 gene was subsequently quantified by RT-qPCR. The displayed value is the mean of n=3 independent experiments. Error bar, s.d.. (**c**) Depletion of Lamtor3 or RagA/B attenuated the AA-stimulated mTORC1 activity. The experiment was conducted as in Fig. 3c. (**d**) Rapamycin and Torin1 inhibited the AA-stimulated mTORC1 activity. The experiment was conducted as in Fig. 3j. Cell lysates were subjected to immuno-blotting for phospho-S6K1 (p-S6K1) and GAPDH.

Supplementary Figure 4

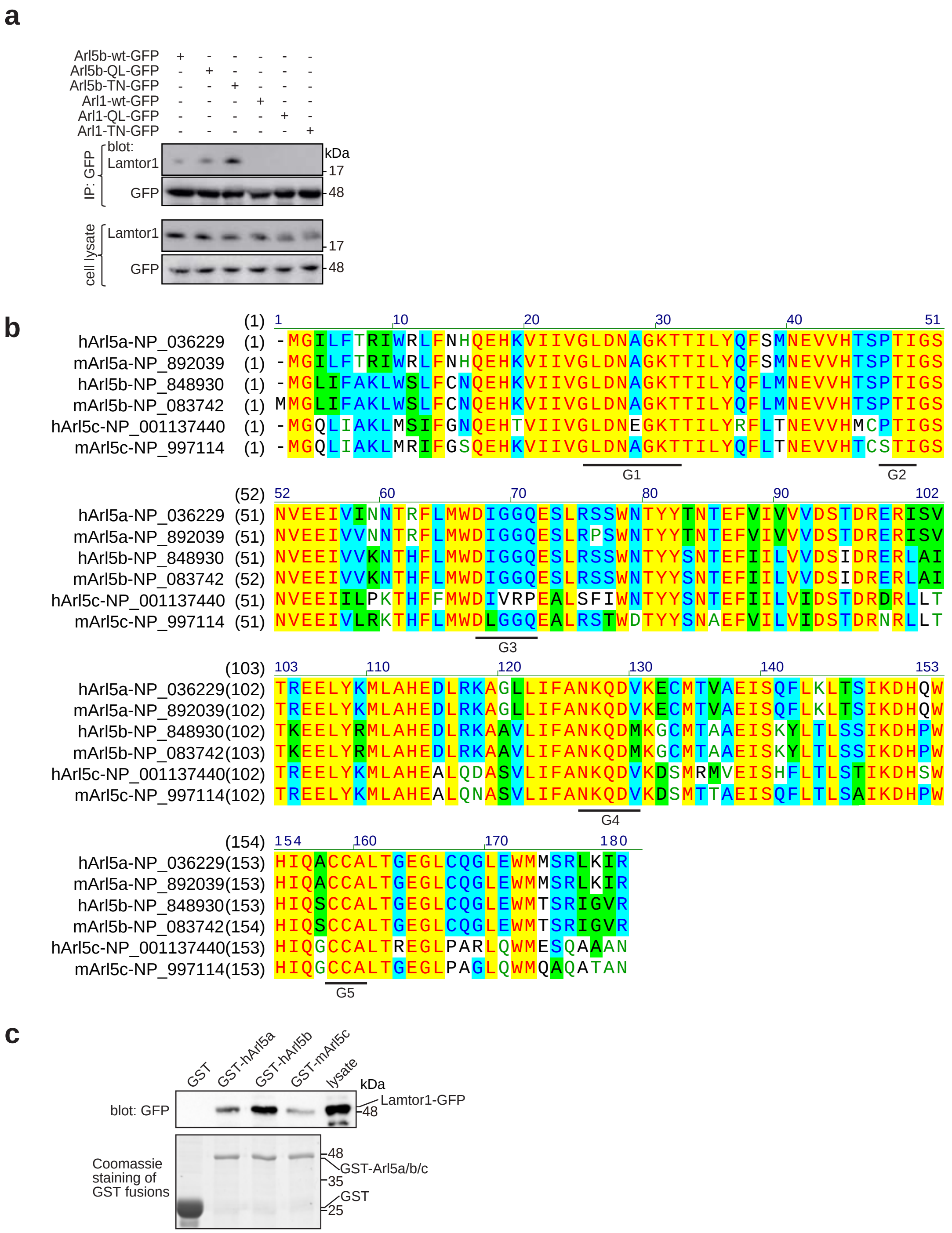

Supplementary Figure 4 Arl5a and b are highly similar in primary sequence and both interact with Lamtor1. (**a**) Arl5b-wt, QL or TN mutant, but not Arl1 corresponding mutant, interacts with Lamtor1. HEK293T cells lysate expressing Arl5b-(wt, QL or TN)-GFP or Arl1-(wt, QL or TN)-GFP were incubated with anti-GFP antibody and IPs were immuno-blotted for endogenous Lamtor1. (**b**) Multiple sequence alignment of human and mouse Arl5a, b and c. The GenBank Accession number of each protein sequence is indicated. The five highly conserved guanine nucleotide binding motifs, G1-5 boxes, are underlined. The protein sequence of hArl5c is significantly different from that of Arl5a, Arl5b or mArl5c, especially at G3. The multiple alignment was conducted in Vector NTI (Invitrogen). (**c**) Human Arl5a and b and mouse Arl5c interact with Lamtor1. Bead-immobilized GST-fusions were incubated with HEK293T cell lysate expressing Lamtor1-GFP and pull-downs were analyzed by immuno-blotting GFP-fusions. The prefix h and m denote human and mouse, respectively.

Supplementary Figure 5

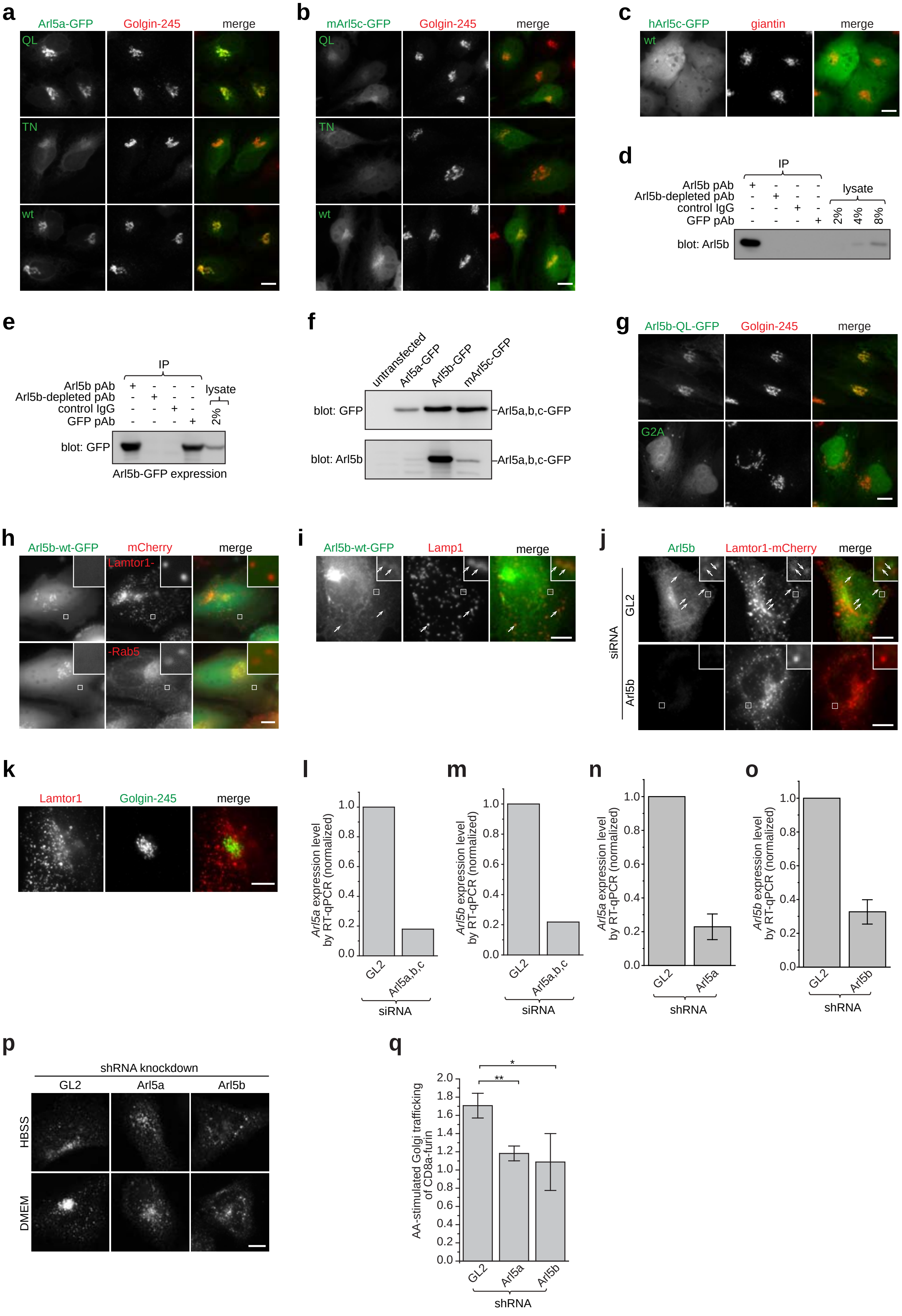

Supplementary Figure 5 Arl5b localization, antibody characterization and Arl5's essential role in the AA-stimulated endosome-to-Golgi trafficking of CD8a-furin. This figure corresponds to Fig. 5. HeLa cells are used. (**a,b,c**) Arl5a and mArl5c, but not hArl5c, localize to the Golgi. Cells transiently expressing indicated GFP-fusions were fixed and endogenous Golgin-245 or giantin was stained. The prefix h and m denote human and mouse, respectively. By default, Arl5 paralogs are from human. (**d,e,f**) Characterization of anti-Arl5b rabbit polyclonal antibody (pAb). In **d**, cell lysates were subjected to co-IP by indicated antibodies and co-IPs were immuno-blotted by anti-Arl5b rabbit pAb. In **e**, cell lysates transiently expressing Arl5b-GFP were subjected to co-IP by indicated antibodies and co-IPs were analyzed by immuno-blotting GFP-fusions. To prepare Arl5b-depleted pAb, Arl5b pAb was incubated with sufficient amount of bead-immobilized GST-Arl5b and the supernatant was used. In **f**, cell lysate transiently expressing GFP-tagged Arl5a, Arl5b or mArl5c was subjected to immuno-blotting by anti-GFP and anti-Arl5b antibody, demonstrating that our anti-Arl5b pAb preferentially recognizes Arl5b. (**g**) N-terminal myristoylation is probably required for the Golgi localization of Arl5b. Cells transiently expressing Arl5b-QL-GFP or Arl5b-QL-GFP harboring G2A mutation were fixed and endogenous Golgin-245 was stained. In Arl5b, Gly at position 2 is a potential myristoylation site. (**h**) Arl5b-wt-GFP does not significantly localize to the endosome or lysosome under live-cell imaging. (**i**) Under methanol fixation, Arl5b-wt-GFP colocalizes with Lamp1 on puncta. Cells expressing Arl5b-wt-GFP were fixed by methanol and processed for immuno-staining of endogenous Lamp1. (**j**) A small pool of endogenous Arl5b specifically colocalizes with Lamtor1. Cells subjected to GL2 or Arl5b siRNA treatment were transfected to express Lamtor1-mCherry. After methanol fixation, cells were processed for immuno-staining of endogenous Arl5b. In **h-j**, the boxed region was enlarged in the upper right corner to show the colocalization at puncta (denoted by arrows). (**k**) Lamtor1 does not localize to the Golgi. Cells were stained for endogenous Lamtor1 and Golgin-245. (**l,m**) Cells were transfected with non-targeting control siRNA (GL2) or a mixture of siRNAs targeting Arl5a, b and c. The transcript expression level of *Arl5a* or *b* gene was quantified by RT-qPCR. The expression level was normalized by that of the corresponding control siRNA. (**n,o**) The knockdown of endogenous Arl5a or Arl5b by lentivirus-mediated transduction of corresponding shRNA. The transcript level was quantified as in **l** and **m**. Data were from n=3 independent experiments. In (**p,q**), After lentivirus-mediated transduction of indicated shRNAs, cells were subsequently transfected to express CD8a-furin and treated with HBSS for 2 h followed by HBSS or DMEM for 20 min before immunofluorescence labeling of CD8a-furin. The AA-stimulated Golgi trafficking of CD8a-furin was calculated for each shRNA. The displayed value is the mean of n=3 independent experiments with each analyzing ≥ 17 cells. Error bar, s.d.; scale bar, 10 µm; *P*-values were from *t*-test; ∗, $P \leq 0.05$; ∗∗, $P \leq 0.005$.

Supplementary Figure 6

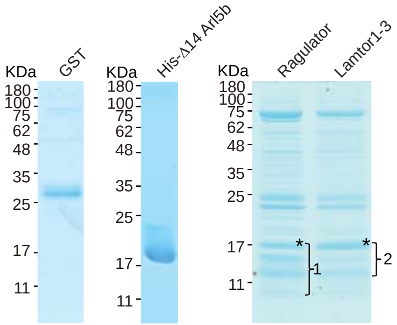

Supplementary Figure 6 Coomassie staining of recombinant proteins used in the *in vitro* guanine nucleotide exchange assay. Molecular weights are labeled on the right of each gel. 1, Lamtor1-5; 2, Lamtor1-3; *, Lamtor1.

Supplementary Figure 7

Fig. 1h

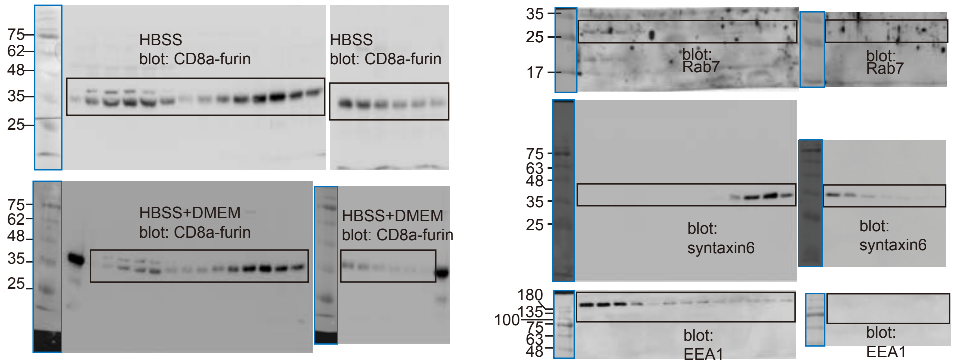

Fig. 3c

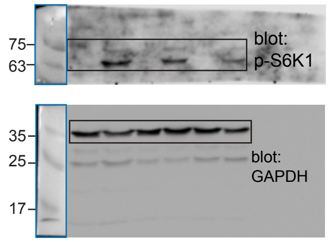

Fig. 3e

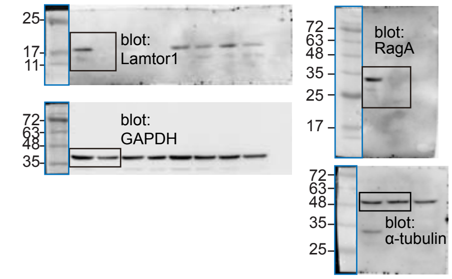

Fig. 3g

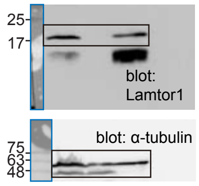

Supplementary Figure 7

Fig. 4a

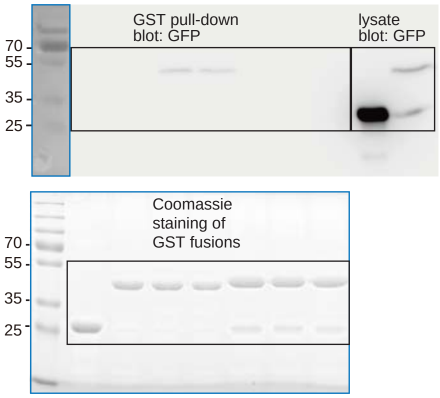

Fig.4b

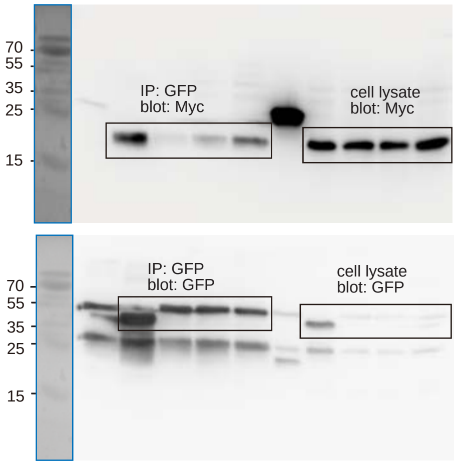

Fig.4c

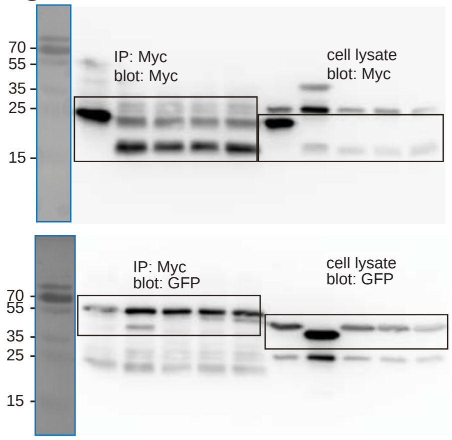

Fig.4d

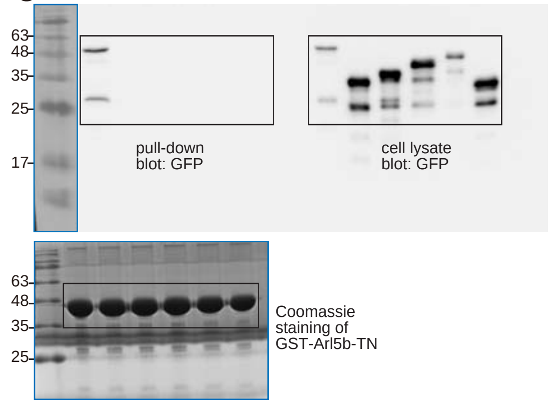

Fig.4e

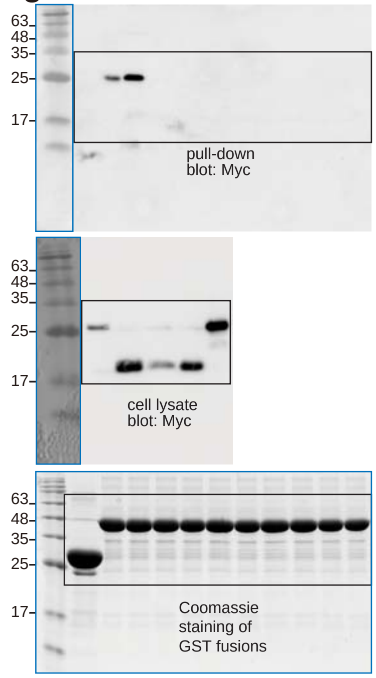

Fig.4f

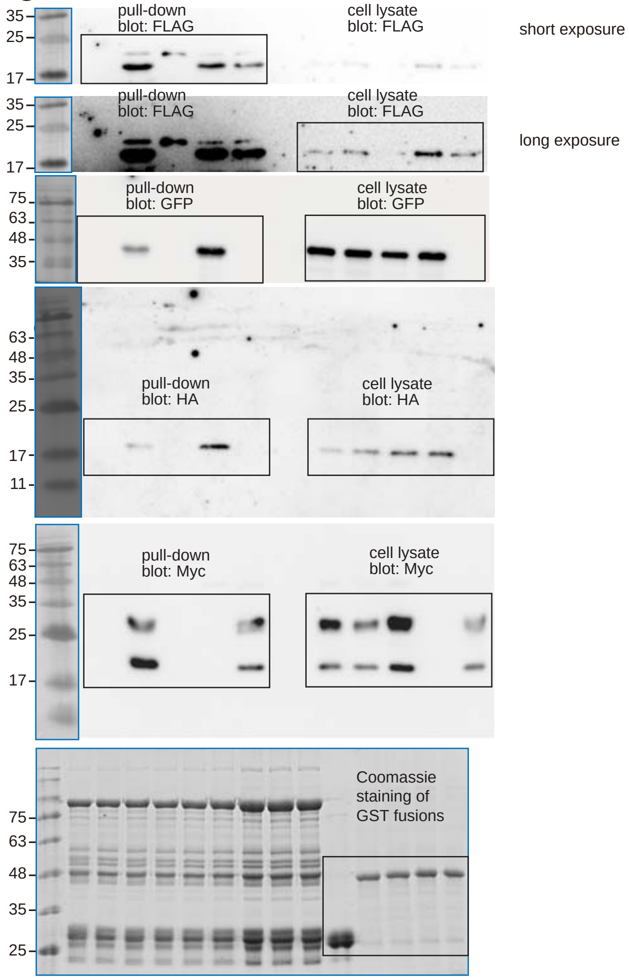

Supplementary Figure 7

Fig.4g

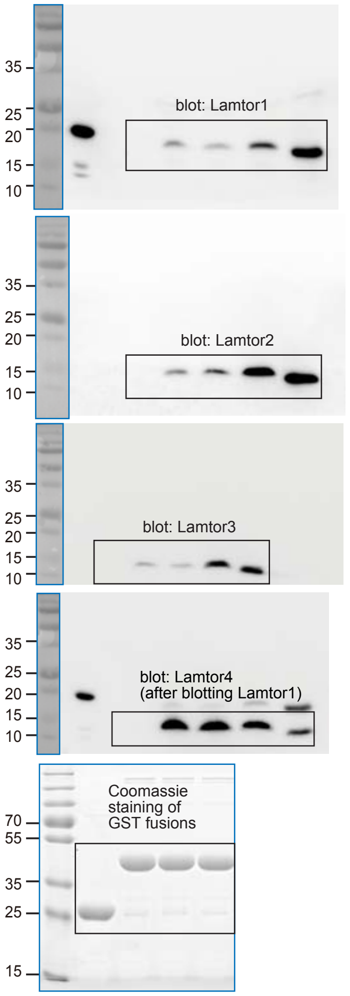

Fig.4h

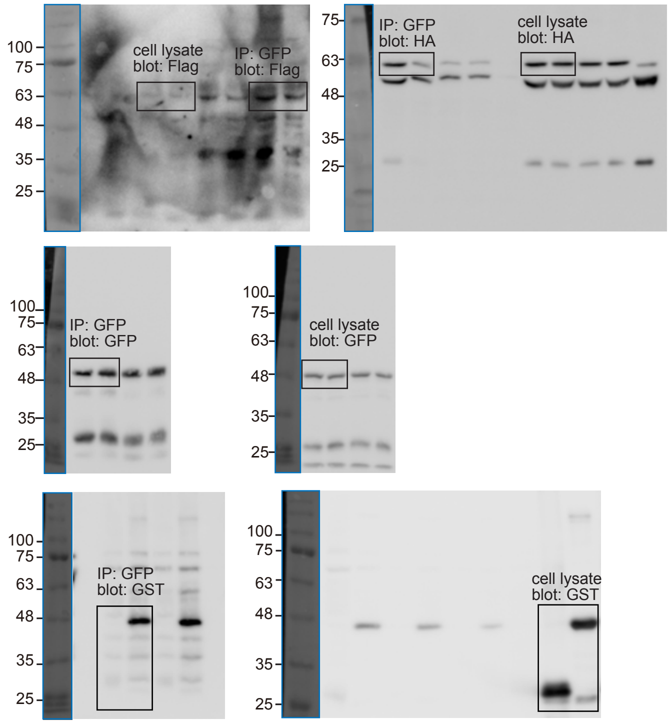

Fig. 5f

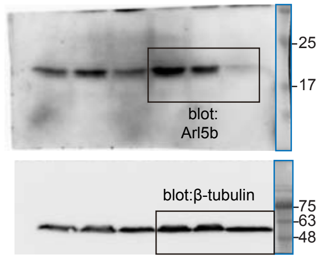

Fig. 5h

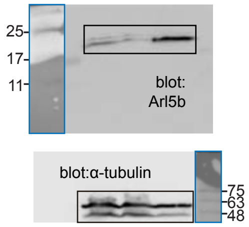

Supplementary Figure 7

Fig.6a

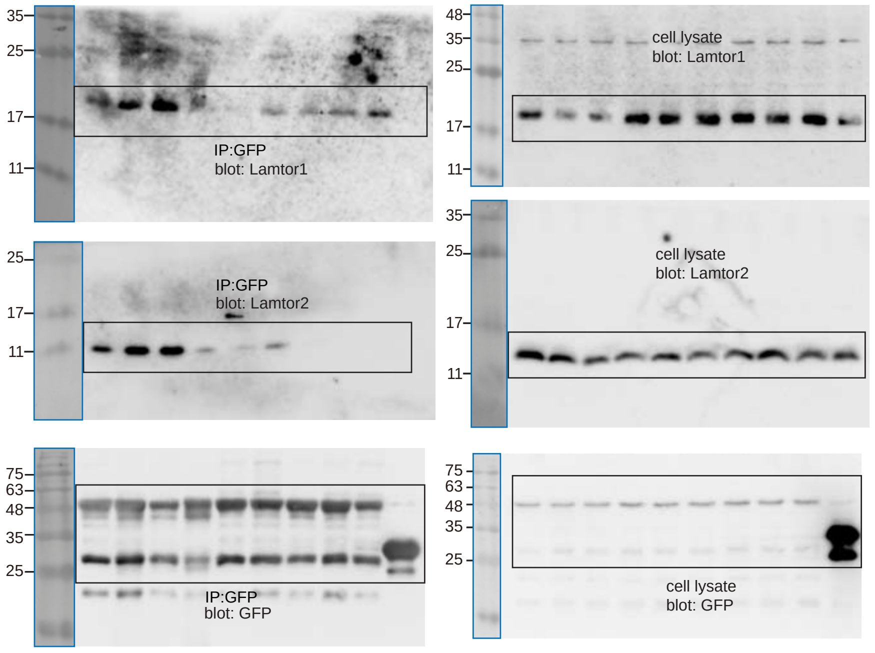

Fig.6b

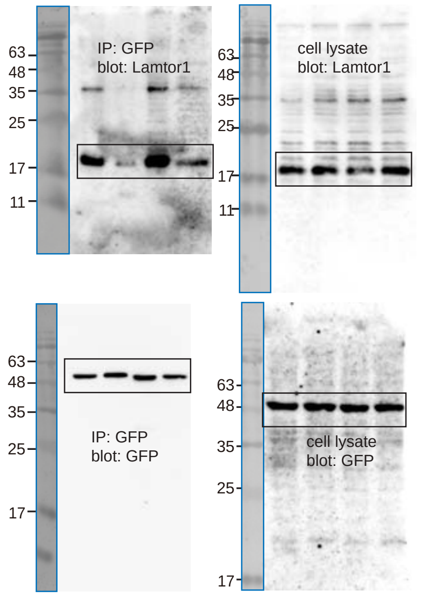

Fig.6c

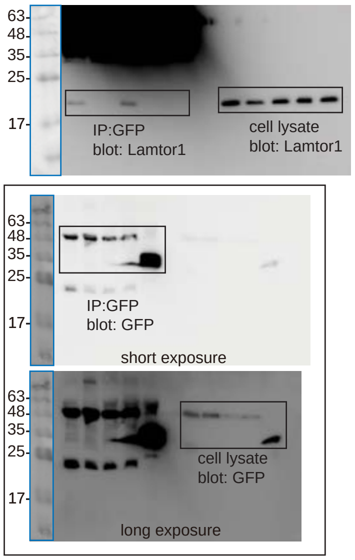

Fig.6d

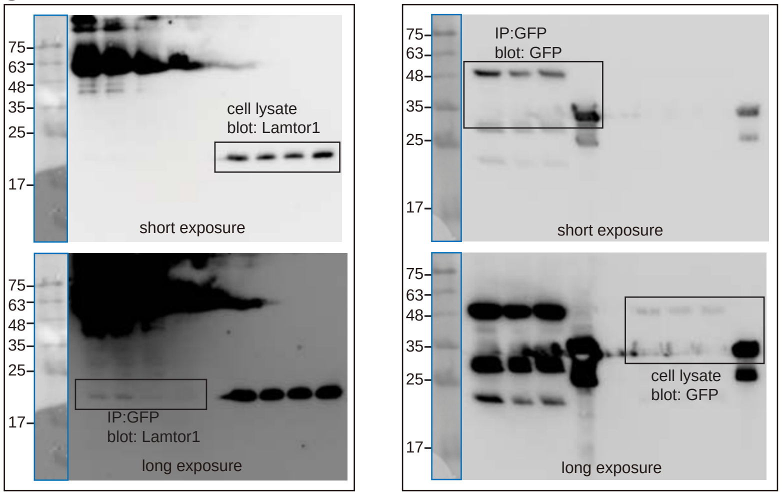

Supplementary Figure 7

Fig.6g

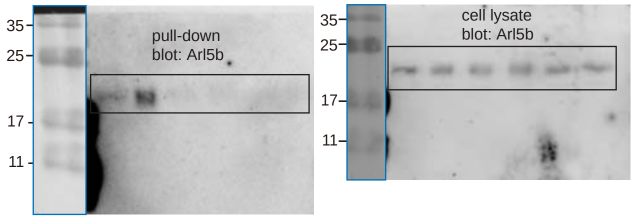

Fig.6h

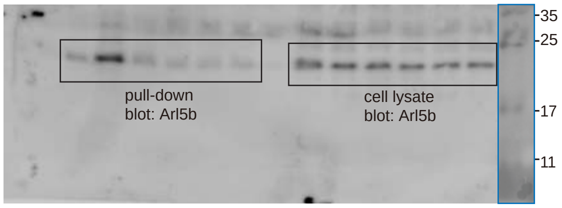

Fig.6i

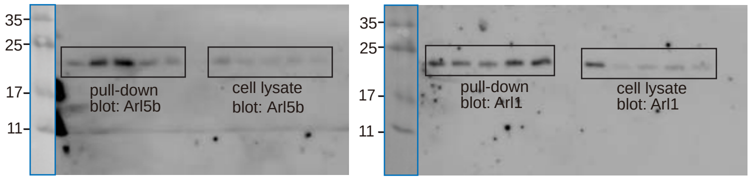

Supplementary Figure 7 Uncropped original gel blot images used in Fig. 1-6. By default, images are chemiluminescence images of blots. White light images showing the molecular weight markers or Coomassie staining of fusion proteins are boxed by blue. Black boxes indicate cropped regions used for the corresponding figure.

Supplementary Methods

DNA plasmids

Constructs of Arl5

To construct Arl5a-GFP, the coding sequence (CDS) of human Arl5a was PCR amplified from a cDNA clone (GenBank Accession No.: NM_012097) using a pair of oligonucleotides (5'-CCG GAA TTC GCC ACC ATG GGA ATT CTC TTC ACT AGA ATA-3' and 5'-CGC GGA TCC CGT CTA ATC TTA AGT CGT GAC ATC-3') as primers and ligated into EcoRI/BamHI digested pEGFP-N1 vector (Clontech) using the same sites. To construct Arl5a-Q70L-GFP, two PCR amplifications were performed using Arl5a-GFP as the template and primer pairs (5'-CCG GAA TTC GCC ACC ATG GGA ATT CTC TTC ACT AGA ATA-3' and 5'-AGA ACG AAG AGA TTC AAG GCC ACC AAT ATC CCA-3') and (5'-TGG GAT ATT GGT GGC CTT GAA TCT CTT CGT TCT-3' and 5'-CGC GGA TCC CGT CTA ATC TTA AGT CGT GAC ATC-3'). The two PCR fragments were mixed and subjected to a second round of PCR amplification using the first and the fourth primer. The resulting PCR product was digested by EcoRI/BamHI and ligated into pEGFP-N1 vector using the same sites. Arl5a-T30N-GFP was similarly constructed using primer pairs (5'-CCG GAA TTC GCC ACC ATG GGA ATT CTC TTC ACT AGA ATA-3' and 5'-TTG GTA AAG AAT GGT AGT TTT CCC TGC ATT ATC-3') and (5'-GAT AAT GCA GGG AAA ACT ACC ATT CTT TAC CAA-3' and 5'-CGC GGA TCC CGT CTA ATC TTA AGT CGT GAC ATC-3'). To construct GST-Arl5a, Arl5a-GFP was digested by EcoRI/BamHI and the released insert was ligated into pGEB vector[1] using the same sites.

To construct Arl5b-GFP, the CDS of human Arl5b was PCR amplified from a cDNA clone (IMAGE clone: 5782580, GenBank Accession No: BQ270027) using a pair of oligonucleotides (5'-CCG GAA TTC GCC ACC ATG GGG CTG ATC TTC GCC AAA CTG TG-3' and 5'-CTA GCT GGA TCC CGT CTC ACA CCA ATC CGG GAG-3') as primers and ligated into EcoRI/BamHI digested pEGFP-N1 vector using the same sites. To construct Arl5b-QL-GFP, two PCR amplifications were performed using Arl5b-GFP as the template and primer pairs (5'-CCG GAA TTC GCC ACC ATG GGG CTG ATC TTC GCC AAA CTG TG-3' and 5'-GAT CGC AGA GAC TCA AGA CCA CCA ATA TCC CAC-3') and (5'-GTG GGA TAT TGG TGG TCT TGA GTC TCT GCG ATC-3' and 5'-CTA GCT GGA TCC CGT CTC ACA CCA ATC CGG GAG-3'). The two PCR fragments were mixed and subjected to a second round of PCR amplification using the first and the fourth primer. The resulting PCR product was then digested by EcoRI/BamHI and ligated into pEGFP-N1 vector using the same sites. Arl5b-TN-GFP was similarly constructed using primer pairs (5'-CCG GAA TTC GCC ACC ATG GGG CTG ATC TTC GCC AAA CTG TG-3' and 5'-GAT AAT GCA GGG AAA AAT ACC ATT CTT TAC C-3') and (5'-GGT AAA GAA TGG TAT TTT TCC CTG CAT TAT C-3' and 5'-CTA GCT GGA TCC CGT CTC ACA CCA ATC CGG GAG-3'). To construct Arl5b-His, the CDS of Arl5b was PCR amplified using a pair of oligonucleotides (5'-ACG ATA AGA TCT GCC ACC ATG GGG CTG ATC TTC GCC AAA C-3' and 5'-AGT TCA AAG CTT TCT CAC ACC AAT CCG GGA GGT CAT CCA C-3') as primers and ligated into BglII/HindIII digested pET30a vector (Novagen) using the same sites. To construct His-Δ14Arl5b, the coding sequence of Arl5b was PCR amplified from Arl5b-GFP using a pair of oligonucleotides (5'-CTA GTC CAT ATG CAA GAA CAC AAA GTA ATT ATA G-3' and 5'-CTA GTC CTC GAG TTC TCA CAC CAA TCC GGG AG-3') as primers and ligated into NdeI/XhoI digested pET30a vector using the same sites. To construct GST-Arl5b-wt, QL or TN, Arl5b-wt, QL or TN-GFP was digested with EcoRI/BamHI and the resulting fragment was ligated into pGEB vector using the same sites. To construct Arl5b-G2A-QL-GFP, PCR amplification was performed using Arl5b-QL-GFP as the template and a pair of oligonucleotides (5'-ACC GCA GAA TTC GCC ACC ATG GCG CTG ATC TTC GCC AAA CTG-3' and 5'-CAT GAC GGA TCC CGT CTC ACA CCA ATC CGG GAG GTC ATC-3') as primers. The resulting PCR product

was digested by EcoRI/BamHI and ligated into pEGFP-N1 vector using the same sites. To construct Arl5b-QL in pGBKT7 vector, Arl5b-QL-GFP was digested by EcoRI/BamHI and the released insert was ligated into pGBKT7 vector (Clontech) using the same sites.

To construct human and mouse Arl5c-GFP, the CDS of human and mouse Arl5c were amplified from two cDNA clones (GenBank Accession No.: NM_001143968 and BC065791.1, respectively) using primer pairs (5'-GCA CCG GAA TTC GCC ACC ATG GGA CAG CTG ATC GCC-3' and 5'-CTA GCT GGA TCC CGG TTA GCA GCG GCC TGA G-3') and (5'-GCG ATC GAA TTC GCC ACC ATG GGA CAG CTG ATA GCC AAG-3' and 5'-CAC TAC GGA TCC CCG TTG GCG GTG GCC TGA GCT TGC AT-3'), respectively. The resulting PCR products were digested with EcoRI/BamHI and inserted into same enzymes digested pEGFP-N1 vector. To construct mArl5c-QL-GFP, two PCR amplifications were performed using mouse Arl5c-GFP as the template and primer pairs (5'-GCG ATC GAA TTC GCC ACC ATG GGA CAG CTG ATA GCC AAG-3' and 5'-GCC TCC AGG CCC CCT AGG TCC CAC ATG-3') and (5'-CAT GTG GGA CCT AGG GGG CCT GGA GGC-3' and 5'-CTA GCT GGA TCC CGT CTC ACA CCA ATC CGG GAG-3'). The two PCR fragments were mixed and subjected to a second round of PCR amplification using the first and the fourth primer. The resulting PCR product was digested by EcoRI/BamHI and ligated into pEGFP-N1 vector using the same sites. mArl5c-TN-GFP, was similarly constructed using primer pairs (5'-GCG ATC GAA TTC GCC ACC ATG GGA CAG CTG ATA GCC AAG-3' and 5'-GAG AAT GGT GTT CTT CCC TGC-3') and (5'-GCA GGG AAG AAC ACC ATT CTC-3' and 5'-CTA GCT GGA TCC CGT CTC ACA CCA ATC CGG GAG-3'). To construct GST-mArl5c, mArl5c-GFP was digested by EcoRI/BamHI and the released insert was ligated into pGEB vector using the same sites.

CD8a chimeras

CD8a-fused furin[2], CI-M6PR[2], CD-M6PR[2] and sortilin[2] in pCI-neo vector (Promega) and retention using selective hooks construct for furin, ss-Strep-KDEL_ss-SBP-GFP-CD8a-furin[3], were previously described. To construct CD8a-furin-mEos2, the fragment encoding CD8a-furin was PCR amplified from CD8a-furin using oligonucleotides (5'- AGA TCT CGA ATT CGA GCC ACC ATG GCC TTA CCA GTG ACC GCC-3' and 5'-ATG GAT CCG TCA GGT CCT CCT CTG AGA TCA GCT TCT GTT CCC AGA GGG CGC TCT GGT CTT-3') as primers. The resulting fragment was digested by EcoRI/BamHI and ligated into pGAGmEos2-N1 vector (Addgene: #46372; a gift from S. Manley) using the same sites.

Constructs of Lamtors

To construct Lamtor1-GFP, the CDS of Lamtor1 was PCR amplified using a full length clone, which was recovered from our yeast-two hybrid screening, as the template and a pair of oligonucleotides (5'-GAC TAG CTC GAG ATG GGG TGC TGC TAC AGC AGC-3' and 5'-GAA CTC GAA TTC GTG GGA TCC CAA ACT GTA CAA CCA G-3') as the primer pair. The resulting PCR product was digested by XhoI/EcoRI and ligated into pEGFP-N1 vector using the same sites. To construct Lamtor1-mCherry, Lamtor1-GFP was digested by XhoI/EcoRI and the insert released was ligated into pmCherry-N1 using the same sites. To construct Lamtor1-Myc, oligonucleotides (5'-AAT TCA GTA CTC AGA ACA AAA ACT CAT CTC AGA AGA GGA TCT GTA AAG C-3' and 5'-GGC CGC TTT ACA GAT CCT CTT CTG AGA TGA GTT TTT GTT CTG AGT ACT G-3') were annealed to generate a fragment encoding Myc-tag and ligated into EcoRI/NotI digested Lamtor1-GFP using the same sites. Lamtor1-Flag was similarly constructed by ligating annealed oligonucleotides (5'-GAT CCA GTA CTC GAC TAC AAA GAC GAT GAC GAC AAG TAA AGC-3' and 5'-GGC CGC TTT ACT TGT CGT CAT CGT CTT TGT AGT CGA GTA CTG-3') to EcoRI/NotI digested Lamtor1-GFP.

To construct GFP-Lamtor1(1-20) and GFP-Lamtor1(1-39), CDSs of 1-20 and 1-39 AAs of Lamtor1 were PCR amplified using Lamtor1-GFP as the template and primer pairs (5'-CAG ATC CGC TAG CGC TAC CGG TCG CCA CCA TGG TG-3' and 5'-GAA CTC GAA TTC GTC ACT TCC GCT CCT CTC GGT CCT GG-3') and (5'-CAG ATC CGC TAG CGC TAC CGG TCG CCA CCA TGG TG-3' and 5'-GAA CTC GAA TTC GTC AGT TGG GCT CGG CTC CAT TGA GAG C-3'), respectively. The resulting fragments were digested by NheI/EcoRI and ligated into pEGFP-C3 vector (Clontech) using the same restriction sites. Similarly, GFP-Lamtor1(1-81) and GFP-Lamtor1(1-121) were constructed by primer pairs (5'-GAA CTC GAA TTC GTC AGT ACT CAT GCT GCT CCA TGC CCT G-3'and 5'-GAC TAG CTC GAG ATG GGG TGC TGC TAC AGC AGC-3') and (5'-GAA CTC GAA TTC GTC AAC TGG CCA GCA CTT GGT GGG GC-3' and 5'-GAC TAG CTC GAG ATG GGG TGC TGC TAC AGC AGC-3'), respectively, using XhoI/EcoRI sites. To construct Lamtor1-G2A-Strep-Myc, which contains G2A mutation, a Strep-tag and Myc tag, the CDS of Lamtor1 was PCR amplified using Lamtor1-GFP as the template and primers (5'-CTA GTC CTC GAG ATG GCA TGC TGC TAC AGC A-3' and 5'-GTC ACT GTC GAC TGT TTT TCG AAC TGC GGG TGG CTC CAC GAT CCA CCT CCC GAT CCA CCT CCG GAA CCT CCA CCT TTC TCG AAC TGC GGG TGG CTC CAT GCT GAT GGG ATC CCA AAC TGT ACA AC-3'). The PCR product was digested by XhoI/SalI and ligated into pMyc-N1 vector [4] using the same restriction sites. To construct Lamtor2-GFP, the CDS of Lamtor2 was PCR amplified from a cDNA clone (GenBank Accession No.: BC024190) using primers (5'-GTA ATG GA ATT CGA GCC ACC ATG CTG CGC CCC AAG GCT TTG-3' and 5'- CA CTA CGG ATC CAA AGA TGC CGC CAC TTG GGT G-3'). The resulting PCR product was digested by EcoRI/BamHI and ligated into pEGFP-N1 vector using the same sites. To construct Myc-Lamtor2, Lamtor2-GFP was digested by EcoRI/NotI to release GFP and the resulting vector was ligated with the annealed oligonucleotides (5'-AAT TCA GTA CTC AGA ACA AAA ACT CAT CTC AGA AGA GGA TCT GTA AAG C-3' and 5'-GGC CGC TTT ACA GAT CCT CTT CTG AGA TGA GTT TTT GTT CTG AGT ACT G-3'). To construct GFP-Lamtor4, the CDS of Lamtor4 was PCR amplified using a cDNA clone (IMAGE clone: 53000314, GenBank Accession No.: BI598677.1) and the following primer pair (5'-GCG ATC GAA TTC ACT TCT GCG CTG ACC CAG GGG CTG-3' and 5'-GCG ATC GGA TCC TCA GAC ATC AAT GGG CTC CCG ACC-3'). The resulting fragment was digested by EcoRI/BamHI and ligated into pEGFP-C2 using the same restriction sites. Lamtor5-GFP was similarly constructed using a cDNA clone (IMAGE clone: 53000314, GenBank Accession No.: BI598677.1) and the following primer pair (5'-GCG ATC GAA TTC GAG CCA GGT GCA GGT CAC CTC GAC-3' and 5'-GCG ATC GGA TCC TCA AGA GGC CAT TTT GTG CAC TGC C-3'). To construct DMyc-Lamtor3, DMyc-Lamtor4 and DMyc-Lamtor5, their CDSs were PCR amplified from a cDNA clone (IMAGE clone: 4808855, GenBank Accession No.: BC026245), GFP-Lamtor4 and GFP-Lamtor5 using primer pairs (5'-CTA GTC GAA TTC AAT GGC GGA TGA CCT AAA GCG A-3' and 5'-CTA CTC GTC GAC TTA AGA AAC TTC CAC AAC TTG TC-3'), (5'-CTA GTC GAA TTC AAT GAC TTC TGC GCT GAC CCA-3' and 5'-CTA CTC GTC GAC TCA GAC ATC AAT GGG CTC CC-3') and (5'-CTT GGA GAA TTC AAT GGA GCC AGG TGC AGG TC-3' and 5'-CTT GTA GTC GAC TCA AGA GGC CAT TTT GTG CAC-3'), respectively. The resulting PCR products were digested by EcoRI/SalI and ligated into the digested pDMyc-neo vector[2] using the same sites, respectively. To construct DHA-Lamtor3, DMyc-Lamtor3 was digested by EcoRI/SalI and the released insert was subsequently ligated into pDHA-neo vector. Both pDMyc-neo and pDHA-neo vectors have tandem or double tags and the same multiple cloning sites.

Lentivirus expression constructs

To construct CD8a-furin in pLVX-puro vector, the fragment encoding CD8a-furin was PCR amplified from CD8a-furin in pCI-neo using oligonucleotides (5'-GTC TAG AAT TCA GCC ACC ATG GCC TTA CCA GTG ACC GCC TTG C-3' and 5'-GAC CTG TCT AGA TTA GAG GGC GCT

CTG GTC TTT GAT AAA GGC G-3') as primers. The resulting fragment was digested by EcoRI/XbaI and ligated into pLVX-puro vector (Clontech) using the same sites.

shRNA constructs

To construct GL2 shRNA in pLKO.1 vector, oligonucleotide (5'-CCG GAA CGT ACG CGG AAT ACT TCG ACT CGA GTC GAA GTA TTC CGC GTA CGT TTT TTT G-3' and 5'-AAT TCA AAA AAA CGT ACG CGG AAT ACT TCG ACT CGA GTC GAA GTA TTC CGC GTA CGT T-3') were annealed and ligated into AgeI/EcoRI digested pLKO.1 vector (Addgene # 10878; a gift from D. Root). shRNAs targeting Arl5a, Arl5b, Vps51 #1, Vps51 #2, Vps54 #1, Vps54 #2, SLC38A9 #1 and SLC38A9 #2 in pLKO.1 vector were similarly constructed using the following oligonucleotide pairs (5'-CCG GAA TGA TCT CTA CTG ACC TCT TCT CGA GAA GAG GTC AGT AGA GAT CAT TTT TTT G-3' and 5'-AAT TCA AAA AAA TGA TCT CTA CTG ACC TCT TCT CGA GAA GAG GTC AGT AGA GAT CAT T-3'), (5'-CCG GAA TAC CTC ACC CTT AGT TCA ACT CGA GTT GAA CTA AGG GTG AGG TAT TTT TTT G-3' and 5'-AAT TCA AAA AAA TAC CTC ACC CTT AGT TCA ACT CGA GTT GAA CTA AGG GTG AGG TAT T-3'), (5'-CCG GAA CCT CTT GAG CAA TAT CCA GCT CGA GCT GGA TAT TGC TCA AGA GGT TTT TTT G-3' and 5'-AAT TCA AAA AAA CCT CTT GAG CAA TAT CCA GCT CGA GCT GGA TAT TGC TCA AGA GGT T-3'), (5'-CCG GAA CGT ATT GAT GTG TTC AGC CCT CGA GGG CTG AAC ACA TCA ATA CGT TTT TTT G-3' and 5'-AAT TCA AAA AAA CGT ATT GAT GTG TTC AGC CCT CGA GGG CTG AAC ACA TCA ATA CGT T-3'), (5'-CCG GAA CAT TGC TCA CCA GAT CTC TCT CGA GAG AGA TCT GGT GAG CAA TGT TTT TTT G-3' and 5'-AAT TCA AAA AAA CAT TGC TCA CCA GAT CTC TCT CGA GAG AGA TCT GGT GAG CAA TGT T-3'), (5'-CCG GAA CCA GCT GAA GTT CTT ATT GCT CGA GCA ATA AGA ACT TCA GCT GGT TTT TTT G-3' and 5'-AAT TCA AAA AAA CCA GCT GAA GTT CTT ATT GCT CGA GCA ATA AGA ACT TCA GCT GGT T-3'), (5'-CCG GGC CTT GAC AAC AGT TCT ATA TCT CGA GAT ATA GAA CTG TTG TCA AGG CTT TTT G-3' and 5'-AAT TCA AAA AGC CTT GAC AAC AGT TCT ATA TCT CGA GAT ATA GAA CTG TTG TCA AGG C-3') and (5'-CCG GCC TCT ACT GTT TGG GAC AGT ACT CGA GTA CTG TCC CAA ACA GTA GAG GTT TTT G-3' and 5'-AAT TCA AAA ACC TCT ACT GTT TGG GAC AGT ACT CGA GTA CTG TCC CAA ACA GTA GAG G-3').

The following shRNA constructs in pLKO.1 were gifts from D. Sabatini. Lamtor1 (Addgene: #26631), Lamtor3 (Addgene: #26632), RagA #1 (Addgene: #30319), RagB #1 (Addgene: #26627).

RNAi-resistant Lamtor1 and Arl5b expressing constructs

To construct RNAi-resistant Lamtor1 in pLVX-puro vector that can rescue Lamtor1-shRNA mediated knockdown, two PCR amplifications were performed to introduce silent mutations in the shRNA target region, by using Lamtor1 as the template and primer pairs (5'-AGT GAT CTC GAG GCC ACC ATG GGG TGC TGC TAC AG-3' and 5'-ATT ATG TTG GAC GCC GTC TTG GCA AG G ATG GAA GAG-3') and (5'-CGG CGT CCA ACA TAA TAG ATG TGT CTG CTG CAG AC-3' and 5'-AGC GAC GAA TTC TCA TGG GAT CCC AAA CTG TAC-3'). The two PCR fragments were mixed and subjected to a second round of PCR amplification using the first and the fourth primer. The resulting PCR product was digested by XhoI/EcoRI and ligated into pLVX-puro vector using the same sites. To construct RNAi-resistant Arl5b in pLVX-puro vector that can rescue Arl5b shRNA mediated knockdown, two PCR amplifications were performed to introduce silent mutations in shRNA targeted region, by using Arl5b as the template and primer pairs (5'-AGT GAT CTC GAG GCC ACC ATG GGG CTG ATC TTC GC-3' and 5'-GAG CTC AGC GTC AGA TAT TTC GAG ATT TCA GCT GC-3') and (5'-ATA TCT GAC GCT GAG CTC AAT TAA GGA TCA TCC-3' and 5'-AGT GAC GAA TTC TTA TCT CAC ACC AAT CCG GG-3'). The two PCR fragments were mixed and subjected to a second round of PCR amplification using the first and

the fourth primer. The resulting PCR product was digested by XhoI/EcoRI and ligated into pLVX-puro vector using the same sites.

Other constructs

To construct furin-GFP or mCherry, the full length CDS of furin was PCR amplified using a cDNA clone (GenBank Accession No.: BC012181.1) as the template and the following oligonucleotides (5'-CAG ATC TCG AGC TCA AGC TTC GAA TTC GCC ACC ATG GAG CTG AGG CCC TGG-3' and 5'-GAT CCC GGG CCC GCG GTA CCG TCG ACC CGA GGG CGC TCT GGT CTT TG-3') as primers. The PCR fragment was digested by EcoRI/SalI and ligated into pEGFP-N1 or pmCherry-N1 vector, respectively, using the same sites. Plasmid DNAs encoding GST-Arl1, Arl1-(wt, TN and QL)-GFP and Myc-SNX3 were previously described[1, 5]. Lamp1-GFP, GFP-Rab7, mCherry-Rab5, TfR-GFP were gifts from T. Kirchhausen. To construct Lamp1-mCherry, mCherry was released from pmCherry-N1 using BamHI/NotI and ligated into BamHI/NotI digested Lamp1-GFP. Flag-pLJM1-RagB-54L and pRK5-HA-GST-RagC were gifts from D. Sabatini (Addgene: #19314 and #19304).

Quantitative mass spectrometry of cell surface proteins

Heavy HeLa cells were labeled in L-lysine- and L-arginine-free DMEM medium (Thermo Fisher Scientific) with 10% dialyzed fetal bovine serum (Thermo Fisher Scientific, #A3382001) supplemented with isotope-labeled heavy L-Lysine: 2HCl (13C6, 15N2) (Lys8) (100 µg ml^{-1}; Cambridge Isotope Laboratories) and L-Arginine: HCl (13C6) (Arg6) (50 µg ml^{-1}; Cambridge Isotope Laboratories). Light Hela cells were grown in normal DMEM with 10% dialyzed fetal bovine serum. Both types of cells were cultured for 9 passages to ensure a complete isotope labeling. In the forward labeling experiment, heavy and light cells grown in 15-cm Petri-dishes were treated with DMEM and HBSS, respectively, for 2 h. Next, cells were washed by ice cold PBS and subjected to surface biotinylation by incubating with 2 mg ml^{-1} EZ-Link Sulfo-NHS-SS-Biotin (Thermo Fisher Scientific) for 30 min on ice. After washing by 100 mM Glycine in PBS, biotinylated cells were lysed with in ice-cold RIPA buffer containing 2 M urea and 1 × cOmplete™ Protease Inhibitor Cocktail (Roche). The two types of lysates were subsequently mixed. After centrifugation in a table top centrifuge, supernatants were incubated with 500 µl pre-washed Streptavidin magnetic beads (Thermo Fisher Scientific) for 2 h at room temperature. Beads were washed by RIPA buffer containing 2 M urea, RIPA buffer containing 2 M urea + 1 M KCl, RIPA buffer containing 2 M urea + 0.1 M sodium bicarbonate. The biotinylated proteins were eluted from beads by boiling in the SDS sample buffer containing 50 mM DTT and 2 mM biotin. After separating eluted proteins by SDS-PAGE, protein lanes were collected for quantitative mass spectrometry analysis. The reverse labeling experiment was conducted similarly except that light and heavy cells were treated with DMEM and HBSS, respectively. All mass spectrometry analysis was performed in triplicate.

Protein identification and quantification were performed using Proteome Discoverer 1.4.1.14 (Thermo Fisher Scientific) together with the Sequest and Mascot search engines. Search parameters included carbamidomethyl at cysteine as static modification; methionine oxidation and Asn and Gln deamidation as dynamic modifications; trypsin and maximum 2 missed cleavages as digestion parameters; 10ppm precursor mass and 0.02Da fragment mass tolerance. The UniProt Knowledgebase (UniProtKB) of human proteins (downloaded on 25 July, 2016, including 70,849 sequences and 23,964,784 residues) was used as a search database with a false discovery rate (FDR) cut-off of ≤ 0.05% both at peptide and protein level. For SILAC quantification Arg6 and Lys8 were set as label modification. The statistical analysis was performed by using all biological and technical replicates. Statistical significance was calculated using Student's t-test and volcano plot was plotted to determine the cut-off level of the data set. The data from the reverse experiment were reversed and combined with those from the forward

experiment during analysis. Proteins were selected based on below cut-offs: *P*-values ≤ 0.05 and $\log_2$(DMEM/HBSS-ratio) ≤ -0.3 or ≥ 0.5.

Supplementary References

1. Lu, L., Horstmann, H., Ng, C. & Hong, W. Regulation of Golgi structure and function by ARF-like protein 1 (Arl1). *J Cell Sci* **114**, 4543-4555 (2001).
2. Mahajan, D. *et al.* Mammalian Mon2/Ysl2 regulates endosome-to-Golgi trafficking but possesses no guanine nucleotide exchange activity toward Arl1 GTPase. *Sci Rep* **3**, 3362 (2013).
3. Tie, H.C. *et al.* A novel imaging method for quantitative Golgi localization reveals differential intra-Golgi trafficking of secretory cargoes. *Mol Biol Cell* **27**, 848-861 (2016).
4. Madugula, V. & Lu, L. A ternary complex comprising transportin1, Rab8 and the ciliary targeting signal directs proteins to ciliary membranes. *J Cell Sci* **129**, 3922-3934 (2016).
5. Lu, L. & Hong, W. Interaction of Arl1-GTP with GRIP domains recruits autoantigens Golgin-97 and Golgin-245/p230 onto the Golgi. *Mol Biol Cell* **14**, 3767-3781 (2003).
