# Supplementary material for: Amino acids stimulate the endosome-to-Golgi trafficking through Ragulator and small GTPase Arl5
Source: Nat Commun. 2018 Nov 26;9:4987. doi: 10.1038/s41467-018-07444-y (PMC6255761; doi:10.1038/s41467-018-07444-y)
Supplement: Supplementary file 3 — Description of Additional Supplementary Files [file 41467_2018_7444_MOESM3_ESM.pdf]

Description of Additional Supplementary Files

File Name: Supplementary Table 1

Description: List of candidate proteins identified from SILAC cell surface proteomics under DMEM or HBSS treatment. Only candidates with P-value ≤ 0.05 are included. Entries with Log2(DMEM/HBSSratio) ≤ -0.3 are shaded orange and those ≥ 0.5 are shaded green. Transmembrane, GPIanchored and secretory proteins are shaded blue (85 in total) and those localized at the Golgi are further colored pink (10 in total), as predicated according to UniProt annotations unless otherwise specified.
